# Short-Term Intensive Avalglucosidase Alfa Regimen in Late-Diagnosed Infantile Pompe Disease: A Case Report

**DOI:** 10.3390/reports9010031

**Published:** 2026-01-20

**Authors:** Vincenza Gragnaniello, Alice Pozza, Chiara Cazzorla, Daniela Gueraldi, Giovanni Di Salvo, Alberto B. Burlina

**Affiliations:** 1 Division of Inherited Metabolic Diseases, Department of Woman’s and Child’s Health, University Hospital of Padua, Via Giustiniani 2, 35128 Padua, Italy; chiara.cazzorla@aopd.veneto.it (C.C.); daniela.gueraldi@aopd.veneto.it (D.G.); alberto.burlina@unipd.it (A.B.B.); 2 Paediatric Cardiology Unit, Department of Woman’s and Child’s Health, University of Padua, Via Giustiniani 2, 35128 Padua, Italy; alice.pozza@aopd.veneto.it (A.P.); giovanni.disalvo@aopd.veneto.it (G.D.S.)

**Keywords:** Pompe disease, hypertrophic cardiomyopathy, hypotonia, enzyme replacement therapy, avalglucosidase alfa, high-dose regimen

## Abstract

**Background and Clinical Significance:** Classic infantile-onset Pompe disease (IOPD) is the most severe form of Pompe disease, manifesting within the first months of life with hypertrophic cardiomyopathy and severe hypotonia. Avalglucosidase alfa is a next-generation recombinant human α-glucosidase that was recently approved for use. Clinical trials, conducted on IOPD patients already treated with alglucosidase alfa, have recommended a dosage ranging from 20 to 40 mg/kg every other week. The optimal dosage for treatment-naïve patients has not yet been established. We present a case of a severe IOPD patient who received a short-term high-dose, high-frequency regimen of avalglucosidase alfa (40 mg/kg/week). **Case Presentation:** The patient, a 3-month-old infant, presented with hypotonia and severe hypertrophic cardiomyopathy (left ventricular mass index (LVMI) of 136 g/m^2^; ejection fraction (EF) of 60%). Treatment with avalglucosidase alfa was initiated at a dose of 40 mg/kg every other week. After two weeks, cardiac function further deteriorated (LVMI of 168 g/m^2^; EF of 46%), so the treatment was intensified to a dose of 40 mg/kg weekly for two months. This resulted in significant clinical, biochemical, and motor improvements without adverse reactions. Following this improvement, the dosage of 40 mg/kg every other week was reinstated. At 18 months of age, the patient demonstrated normal motor development, normal cardiac function (LVMI of 49 g/m^2^; EF of 68%), and normal biomarkers. **Conclusions:** Although limited to a single patient, this case illustrates that short-term high-dose, high-frequency administration of avalglucosidase alfa could be both effective and safe, even in patients with severe, late-diagnosed IOPD.

## 1. Introduction

Pompe disease (OMIM #232300), also known as glycogen storage disease type II, is an autosomal recessive lysosomal storage disorder. It is caused by a deficiency of acid alpha-glucosidase (GAA), leading to glycogen accumulation in lysosomes of various cell types and tissues, particularly affecting cardiac, skeletal, and smooth muscle cells [1,2].

Classic infantile-onset Pompe disease (IOPD) is the most severe form. It typically presents at birth or within the first few months of life, characterized by hypotonia, feeding difficulties, and respiratory problems. A hallmark of IOPD is hypertrophic cardiomyopathy (HCM), which may develop in utero. Without treatment, patients usually succumb to cardiorespiratory failure within the first year of life [3].

Enzyme replacement therapy (ERT) with recombinant human GAA is currently the only approved treatment. Alglucosidase alfa, the first ERT, was approved in 2006 and has shown effectiveness in improving or stabilizing the disease course. Given the heterogeneity of the disease, the effect is variable. Approved at a dosage of 20 mg/kg every other week, real-world experience has since demonstrated that higher dosages of up to 40 mg/kg weekly are more effective in IOPD patients [4]. Key factors influencing treatment outcomes include age at ERT initiation [5,6], severity of clinical presentation, and cross-reactive immunological material (CRIM) status [7,8,9,10].

However, the efficacy of alglucosidase alfa is limited by poor targeting to skeletal muscles. As the mannose-6-phosphate receptor is crucial for cellular internalization of exogenous lysosomal enzymes [11], new treatments with higher levels of mannose-6-phosphate (M6P) are being developed to enhance enzyme uptake.

Avalglucosidase alfa represents a next-generation recombinant human α-glucosidase (neorhGAA). It is conjugated with multiple synthetic bis-mannose-6-phosphate (bis-M6P)-tetra-mannose glycans, improving muscle cell uptake and glycogen clearance [12,13]. This treatment received approval from the Food and Drug Administration (FDA) in 2021 and the European Medicines Agency (EMA) in 2022 [11,14].

The standard dosage for avalglucosidase alfa is 20 mg/kg body weight every other week. For IOPD patients showing insufficient improvement in cardiac, respiratory, and/or motor function at this dose, an increase to 40 mg/kg every other week may be considered [1]. Clinical trials and real-world data have already demonstrated the efficacy of high dosage in IOPD patients previously treated with alglucosidase alfa [4,15]. The optimal dosage for treatment-naïve IOPD patients has not yet been established, and real-world data are lacking.

We present a case of an IOPD patient diagnosed at 3 months of age, presenting with severe HCM and hypotonia. A high-dose, high-frequency regimen of avalglucosidase alfa (40 mg/kg/week) was initiated, resulting in positive outcomes without adverse events.

## 2. Case Report

The patient, a female, was the third child of non-consanguineous Italian parents. She was born at term following a pregnancy complicated by maternal insulin-dependent diabetes mellitus. Prenatal ultrasound examinations had not revealed any concerns. At birth, she was classified as large for gestational age (birth weight of 3680 g, 90th percentile; length of 53 cm, 99th percentile; head circumference of 35.5 cm, 95th percentile; all according to CDC). Apgar scores were 8-8-8 at 1, 5, and 10 min.

Perinatally, the infant experienced respiratory distress requiring non-invasive respiratory support. An electrocardiogram (ECG) showed a short PR interval (80 msec) and high-voltage QRS complexes. Echocardiography (Echo) revealed concentric HCM with initial diastolic dysfunction without outflow obstruction. These findings were initially attributed to maternal diabetes mellitus. Extended newborn screening was performed, but lysosomal storage disorders were not included in the screening panel in her Italian region of birth. Her cardiac status remained stable in the first months of life, but she presented with axial hypotonia and delayed psychomotor development.

At 3 months, she was referred to our center for inborn errors of metabolism. Upon admission, clinical examination revealed a floppy infant phenotype (Alberta Infant Motor Scale (AIMS) score 0, <5 pc, performed by an experienced pediatric neuropsychiatrist—DG). Oxygen saturation ranged from 92 to 94%, with mild tachypnea (45–50 breaths per minute) and respiratory distress. Nocturnal desaturation < 90% required supplemental oxygen. ECG findings included a PR interval of 80 ms, left axis deviation, high voltage across leads, and repolarization abnormalities. Echo was performed by one pediatric cardiologist (AP) using a Vivid E95 Ultrasound machine (GE Vingmed Ultrasound AS, Horten, Norway) with full assessment of cardiac structure and function according to current guidelines [16], and it was reviewed by a single experienced reader (GDS). Echo confirmed HCM, with an increased left ventricular mass index (LVMI) of 136 g/m^2^ (normal reference value <84 g/m^2^, according to the Boston Children’s Hospital Z-score system). Additional findings included markedly reduced longitudinal function, with initial spongy appearance in the mid-apical region, along with preserved global systolic function (ejection fraction (EF) of 60%; global longitudinal strain (GLS) of −10%).

Blood tests showed elevated muscle enzymes (CPK 660 U/L, ALT 158 U/L, AST 262 U/L, and LDH 1272 U/L). GAA enzymatic activity on dried blood spots was reduced (0.10 µM/h; normal range, 2.3–15). Pompe disease was confirmed by GAA enzymatic activity in lymphocytes (0.08 µmol/mg/h; normal range, 5.48–40) and elevated urinary tetrasaccharide (uGlc4) levels (24.5 mmol/mol creatinine; normal range, 0–16.3 for age). Western blot analysis indicated CRIM positivity, which was subsequently confirmed by molecular testing of the *GAA* gene, revealing compound heterozygosity for variants c.1438-2A>G and c.1465G>A (p.Asp489Asn), both classified as pathogenic (ACMG class 5). The two variants were confirmed to be in trans by familial segregation.

**Figure 1:**
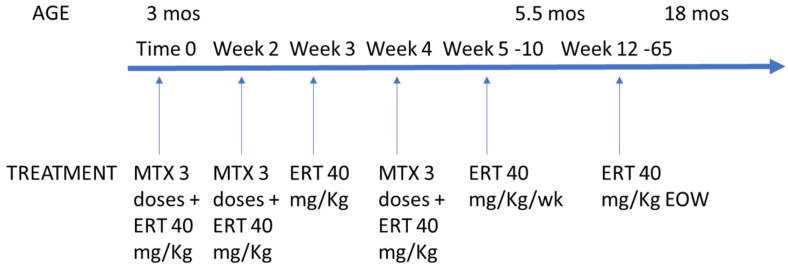
Timeline of enzymatic replacement therapy and immunomodulation. MTX: methotrexate; ERT: enzyme replacement therapy.

The patient immediately began immune tolerance induction with transient low-dose methotrexate and ERT with avalglucosidase alfa (40 mg/kg every other week). The methotrexate protocol involved subcutaneous administration at 0.4 mg/kg for three consecutive days, with the initial dose given 15 min prior to ERT initiation. This regimen was repeated in three cycles, each separated by a two-week interval, following the protocol described by Kazi et al. [17] (Figure 1).

After 2 weeks, motor condition remained compromised, and cardiac status worsened. Echo showed increased LVMI (168 g/m^2^) and worsening of global function (EF 46%; GLS −11.9%), with marked trabeculation in the mid-apical region and interventricular septal dyskinesia.

Although a single infusion was insufficient to establish therapeutic efficacy, the clinical course indicated disease progression according to its natural history. Cardiac deterioration, persistent respiratory distress, and severe hypotonia necessitated the improvement of the therapeutic approach in a short time. We therefore maximized the etiological therapy with a high-dose, high-frequency avalglucosidase alfa regimen (40 mg/kg/week) based on what had already been demonstrated with alglucosidase alfa [4].

After 2 months of high-dose, high-frequency therapy (age 5.5 months), the patient showed only mild axial and appendicular hypotonia. Head control was good in pull-to-sit and supine positions. She sat with support, grasped objects, and brought them to the midline. Anti-gravity movements were present during ventral suspension (AIMS score 15, 10 pc). She no longer required nocturnal oxygen support, and her respiratory dynamics improved. Echo showed improvement in LVMI (96 g/m^2^), though global function as assessed by EF reduced, but GLS showed a marked improvement (EF 33%; GLS −14.2%). PR interval on ECG was 90 ms. Biomarkers achieved the normal range (CPK 222 U/L; uGlc4 5.9 mmol/mol creatinine). As clinical benefit was achieved and biomarkers normalized, the frequency of therapy with avalglucosidase at a dose of 40 mg/kg was reduced to every 2 weeks in accordance with the drug’s datasheet. A transient increase in uGlc4 was observed after 2 weeks (17.4 mmol/mol creatinine), which normalized again after 3 months.

When the patient was 9 months old and had already resumed the therapeutic regimen of 40 mg/kg EOW for 3.5 months, we measured GAA activity on dried blood spot after 1 week (5.57 μM/h, nv 2.3–15) and 2 weeks (1.52 μM/h, nv 2.3–15) from the previous administration. This confirms that after 1 week, the amount of enzyme in the blood decreases; however, in the absence of pharmacokinetic data on large populations, we continued to make decisions based on the patient’s clinical course.

**Figure 2:**
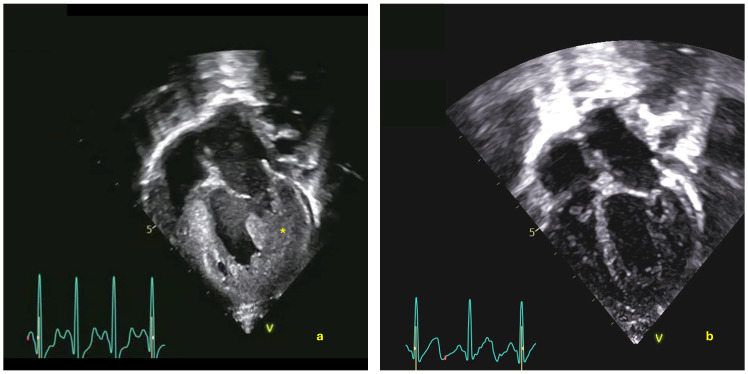
Two-dimensional echocardiogram images, four-chamber apical view. (**a**) Echocardiogram at presentation showing left ventricle hypertrophy (*) and dilatation. (**b**) Echocardiogram after avalglucosidase alfa therapy at 12 months with significant reduction in LV hypertrophy.

At 12 months of life, the patient could stand without support and walk with support (AIMS score 53, 50 pc). Left ventricular hypertrophy improved, with normalization of the LVMI (61 g/m^2^). Both global and longitudinal function showed significant improvement (EF 63%; GLS −18.5) (Figure 2). PR interval on ECG was 100 ms. Biomarkers remained negative (CPK 172 U/L, uGLC4 5.1).

**Figure 3:**
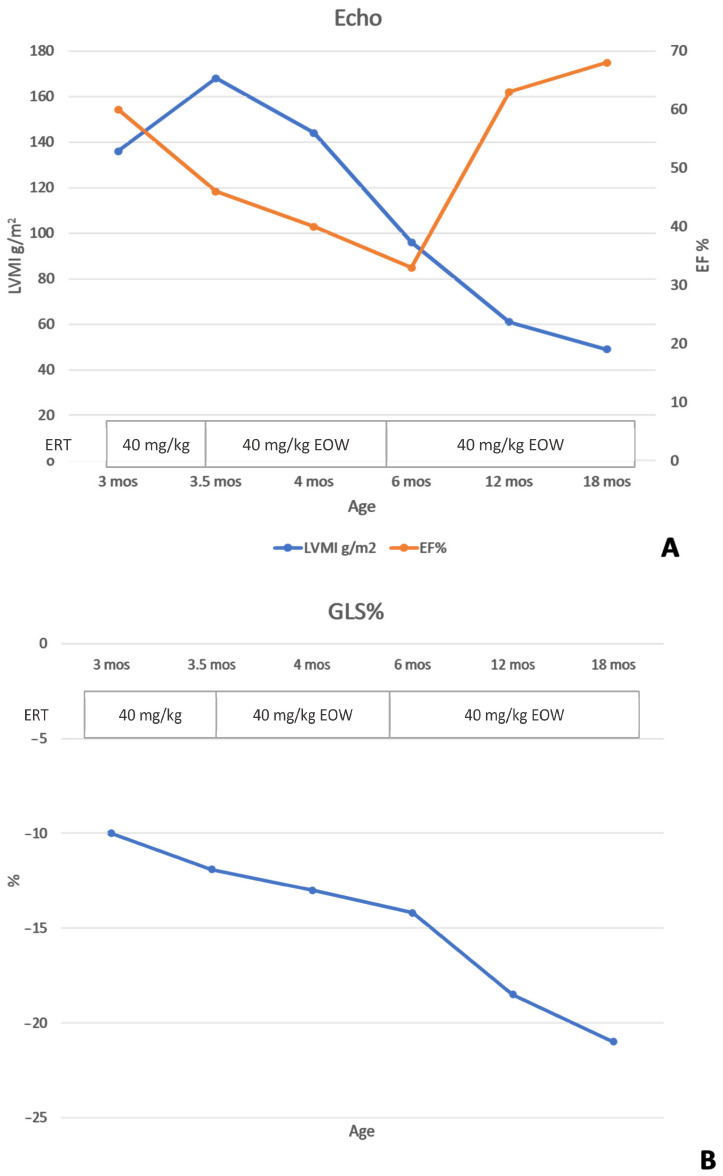
Cardiac parameters over time. Graph shows effect of avalglucosidase alfa therapy over infant’s age expressed in months (mos), both on heart function and cardiac mass remodeling. (**A**) Left ventricle mass index (LVMI) is represented by blue line; ejection fraction (EF) is represented by orange line. Both variables are plotted versus time and depict progressive improvement over time. (**B**) Myocardial deformation of infantile onset Pompe disease over time, showing progressive improvement of global longitudinal strain (GLS) values.

**Figure 4:**
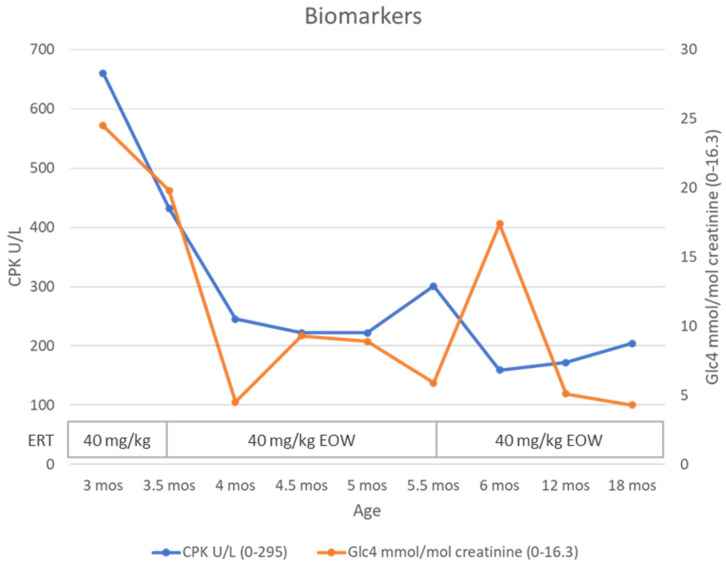
Biomarkers over time. CPK (U/L) and urinary tetrasaccharide (uGlc4, mmol/mol creatinine) over time, showing rapid improvement after start of high-dose, high-frequency therapy.

At 18 months of life, the patient walks without support and climbs and descends stairs with support (AIMS score 60, 90 pc). Echo showed further improvement with normalization of all parameters (LVMI 49 g/m^2^, EF 68%, and GLS −21) (Figure 3). PR interval on ECG was 115 ms. Biomarkers remained negative (CPK 204 U/L; uGLC4 4.3) (Figure 4). IgG antibodies against rhGAA and neo-GAA (LabCorp, Burlington, NC, USA) were monitored monthly for the first 6 months, then every 3 months, and remained negative. The patient did not present hypersensitivity reactions, renal problems related to protein load, or metabolic derangements.

## 3. Discussion

We report a case of classic infantile-onset Pompe disease in which a treatment-naive patient received short-term high-dose, high-frequency avalglucosidase alfa therapy. To our knowledge, this is the first case reported with this treatment dosage regimen.

After 2 months of high-dose treatment, our patient showed normalization of motor function and biomarkers and an improvement in cardiac function that subsequently normalized. The patient has maintained this benefit to date, after 18 months of follow-up.

Although the outcome of treatment-naive patients with avalglucosidase alfa has not yet been reported, the course of our patient differs from what has been reported so far for patients treated with alglucosidase alfa [4,15,18].

The largest Italian experience is reported by Parini et al., who described the outcomes of 28 IOPD patients (70.8% CRIM positive) treated with alglucosidase alfa at a dose of 20 or 40 mg/kg EOW, with a median age of therapy initiation at 4 months. Nine patients died within the first 20 months of life. Only seven achieved independent ambulation at a median age of 16.5 months. Cardiac parameters normalized only in 15 patients at a median age of 12 months [18].

Internationally, the largest real-world IOPD cohort was reported by Ditters et al., who described 124 European patients, of whom 116 were treated with ERT at various dosages, with a median age of therapy initiation of 3.3 months and a mean follow-up of 60.1 months. A total of 41% started ERT at a higher dosage than the standard recommended dosage. The authors concluded that high-dosage, high-frequency ERT regimen (40 mg/kg/week) would optimize glycogen clearance from cells and clinical outcomes in patients with IOPD [4].

Moreover, the time when therapy is started is important. Previous studies on newborn screening have also demonstrated that benefits were achieved when therapy was initiated early. Delays of even a few days can influence outcomes [19,20]. In our experience, we identified, through newborn screening, three IOPD patients treated between days 5 and 19 of life with alglucosidase alfa at a dose of 40 mg/kg/weekly. Two showed an excellent response, with normalization of cardiac mass, normal acquisition of psychomotor development milestones, and negative biomarkers within the first year of life; in one patient, unfortunately, the course was complicated by the development of high-titer sustained anti-ERT antibodies, and the outcome was worse [5].

While with alglucosidase alfa, extensive real-world data already shows that both high-dosage and high-frequency treatments optimize clinical outcome in patients with IOPD, avalglucosidase alfa is a recently approved drug, with most data being derived from clinical studies. The Mini-COMET study, a phase 2 cohort study, evaluated its safety and efficacy in long-term IOPD survivors aged 1 to 12 years who were incompletely responsive to alglucosidase alfa. The highest dose tested, 40 mg/kg every other week, appeared to offer additional benefits in meaningful outcome measures while maintaining a favorable safety profile and acceptable immunogenicity compared to the dosage of 20 mg/kg every other week [21,22].

These findings were corroborated by an Italian real-life study of four IOPD patients (aged 6 to 16 years, mean age 12.25 years) who were experiencing clinical decline on alglucosidase alfa treatment at 40 mg/kg/week. They received avalglucosidase alfa through a compassionate use program. Two patients started at a dose of 20 mg/kg every other week, increasing to 40 mg/kg every other week after 8–10 infusions, while the other two received 40 mg/kg every other week from the start. Avalglucosidase alfa was well-tolerated in all patients with no adverse events reported. Despite varied outcomes due to the real-world setting, the findings suggest a significant positive effect, with constant and rapid decreases in biomarkers and improvements in motor performance. Surprisingly, even cardiac involvement showed improvement in one patient with hypertrophic cardiomyopathy at baseline [23]. Similar results were obtained from a real-world study on nine patients conducted in East Asia [24].

However, data on treatment-naive patients are still lacking both from trials and real-world studies.

In our treatment-naive IOPD patient, we employed a high-frequency and high-dosage regimen due to severe cardiac and motor impairment and late diagnosis at 3 months of age. Although cardiac deterioration at the beginning of therapy could represent the natural trajectory of the disease, previous studies have demonstrated that there is a window in which cardiac improvement is achievable.

A previous report by Chen Lei-Ru et al. demonstrates that cardiac remodeling can be unpredictable after 5 months of age, particularly in the presence of systolic dysfunction, and these patients seem to have a less favorable prognosis. This is speculated to be due to a loss of myofibrils caused by glycogen accumulation in the cytosol, resulting in systolic dysfunction [25]. Our previous experience with an IOPD patient with persistent hypertrophic cardiomyopathy who switched from alglucosidase alfa to a new-generation ERT at 2.5 years of age (cipaglucosidase alfa + miglustat) confirms this finding. Indeed, the benefit on the cardiac aspect had been remarkable with a high weekly dosage of ERT (30 mg/kg/week), but it had evolved into non-compaction cardiomyopathy, probably due to late cardiac remodeling [26].

Given the severity of systolic dysfunction noted in our patient at only 3 months of age, we opted for aggressive treatment to promote maximal cardiac remodeling before irreversible muscle damage could occur.

The high dosage was supported by previous pharmacokinetic studies, which demonstrated that avalglucosidase alfa exposure increased dose-proportionally without apparent deviation [27]. Moreover, pediatric patients eliminate recombinant GAA faster than adults. As the dose is increased to 40 mg/kg every other week in children, AUC values approach those in adults on standard doses. For these reasons, pediatric patients require higher dosing regimens to match the pharmacokinetics of recombinant GAA seen in adults [14]. However, comparative pharmacokinetic data on different administration frequencies are not currently available.

In our patient, the short-term intensive avalglucosidase alfa regimen proved to be safe and well-tolerated, and the patient maintains the benefits achieved at 18 months of life even after returning to the standard dosage of 40 mg/kg EOW. If the clinical course should prove unsatisfactory, a high-frequency administration regimen may be reconsidered.

Despite the favorable outcome, we are nevertheless aware of the limitation associated with reporting a single clinical case, and the fact that multiple factors may have contributed to this outcome, particularly the CRIM-positive status and the immunomodulation protocol [28], in addition to the high-dosage and high-frequency treatment regimen.

While larger patient studies are necessary, it is possible that higher dosages may be used in real-life settings. This was previously observed with alglucosidase alfa, which was initially approved at a dosage of 20 mg/kg every other week but later used with a wide range of dosages [4].

## 4. Conclusions

In conclusion, we report a single case of a naive IOPD patient treated with a short-term high-dose, high-frequency avalglucosidase alfa regimen. While larger studies are necessary to confirm safety and efficacy, our experience suggests that early aggressive treatment with higher doses and frequency could be beneficial in preventing irreversible muscle and cardiac damage in severely affected IOPD patients.

## Data Availability

The original contributions presented in this study are included in the article. Further inquiries can be directed to the corresponding author.

## References

[1] Parenti G., Fecarotta S., Alagia M., Attaianese F., Verde A., Tarallo A., Gragnaniello V., Ziagaki A., Guimaraes M.J., Aguiar P. (2024). The European Reference Network for Metabolic Diseases (MetabERN) Clinical Pathway Recommendations for Pompe Disease (Acid Maltase Deficiency, Glycogen Storage Disease Type II). Orphanet J. Rare Dis..

[2] van der Ploeg A.T., Reuser A.J. (2008). Pompe’s disease. Lancet.

[3] Gupta N., Kazi Z.B., Nampoothiri S., Jagdeesh S., Kabra M., Puri R.D., Muranjan M., Kalaivani M., Rehder C., Bali D. (2020). Clinical and Molecular Disease Spectrum and Outcomes in Patients with Infantile-Onset Pompe Disease. J. Pediatr..

[4] Ditters I.A.M., Huidekoper H.H., Kruijshaar M.E., Rizopoulos D., Hahn A., Mongini T.E., Labarthe F., Tardieu M., Chabrol B., Brassier A. (2022). Effect of Alglucosidase Alfa Dosage on Survival and Walking Ability in Patients with Classic Infantile Pompe Disease: A Multicentre Observational Cohort Study from the European Pompe Consortium. Lancet Child Adolesc. Health.

[5] Gragnaniello V., Pijnappel P.W.W.M., Burlina A.P., In’t Groen S.L.M., Gueraldi D., Cazzorla C., Maines E., Polo G., Salviati L., Di Salvo G. (2022). Newborn Screening for Pompe Disease in Italy: Long-Term Results and Future Challenges. Mol. Genet. Metab. Rep..

[6] Yang C.F., Liao T.E., Chu Y.L., Chen L.Z., Huang L.Y., Yang T.F., Ho H.-C., Kao S.-M., Niu D.-M. (2023). Long-term outcomes of very early treated infantile-onset Pompe disease with short-term steroid premedication: Experiences from a nationwide newborn screening programme. J. Med. Genet..

[7] Kishnani P.S., Corzo D., Leslie N.D., Gruskin D., Van Der Ploeg A., Clancy J.P., Parini R., Morin G., Beck M., Bauer M.S. (2009). Early Treatment With Alglucosidase Alfa Prolongs Long-Term Survival of Infants With Pompe Disease. Pediatr. Res..

[8] Kishnani P.S., Goldenberg P.C., DeArmey S.L., Heller J., Benjamin D., Young S., Bali D., Smith S.A., Li J.S., Mandel H. (2010). Cross-Reactive Immunologic Material Status Affects Treatment Outcomes in Pompe Disease Infants. Mol. Genet. Metab..

[9] Li C., Desai A.K., Gupta P., Dempsey K., Bhambhani V., Hopkin R.J., Ficicioglu C., Tanpaiboon P., Craigen W.J., Rosenberg A.S. (2021). Transforming the Clinical Outcome in CRIM-Negative Infantile Pompe Disease Identified via Newborn Screening: The Benefits of Early Treatment with Enzyme Replacement Therapy and Immune Tolerance Induction. Genet. Med..

[10] Gragnaniello V., Deodato F., Gasperini S., Donati M.A., Canessa C., Fecarotta S., Pascarella A., Spadaro G., Concolino D., Burlina A. (2022). Immune Responses to Alglucosidase in Infantile Pompe Disease: Recommendations from an Italian Pediatric Expert Panel. Ital. J. Pediatr..

[11] Dhillon S. (2021). Avalglucosidase Alfa: First Approval. Drugs.

[12] Pena L.D.M., Barohn R.J., Byrne B.J., Desnuelle C., Goker-Alpan O., Ladha S., Laforêt P., Mengel K.E., Pestronk A., Pouget J. (2019). Safety, Tolerability, Pharmacokinetics, Pharmacodynamics, and Exploratory Efficacy of the Novel Enzyme Replacement Therapy Avalglucosidase Alfa (neoGAA) in Treatment-Naïve and Alglucosidase Alfa-Treated Patients with Late-Onset Pompe Disease: A Phase 1, Open-Label, Multicenter, Multinational, Ascending Dose Study. Neuromuscul. Disord..

[13] Zhu Y., Jiang J.-L., Gumlaw N.K., Zhang J., Bercury S.D., Ziegler R.J., Lee K., Kudo M., Canfield W.M., Edmunds T. (2009). Glycoengineered Acid α-Glucosidase With Improved Efficacy at Correcting the Metabolic Aberrations and Motor Function Deficits in a Mouse Model of Pompe Disease. Mol. Ther..

[14] Punnoose A.R., Jeng L.J.B., Maynard J.W., Review Team (2022). Regulatory News: Avalglucosidase Alfa-Ngpt (Nexviazyme) for Late-Onset Pompe Disease-FDA Approval Summary. J. Inherit. Metab. Dis..

[15] Spada M., Pagliardini V., Ricci F., Biamino E., Mongini T., Porta F. (2018). Early higher dosage of alglucosidase alpha in classic Pompe disease. J. Pediatr. Endocrinol. Metab..

[16] Lopez L., Frommelt P.C., Colan S.D., Trachtenberg F.L., Gongwer R., Stylianou M., Bhat A., Burns K.M., Cohen M.S., Dragulescu A. (2021). Pediatric Heart Network Echocardiographic Z Scores: Comparison with Other Published Models. J. Am. Soc. Echocardiogr..

[17] Kazi Z.B., Desai A.K., Troxler R.B., Kronn D., Packman S., Sabbadini M., Rizzo W.B., Scherer K., Abdul-Rahman O., Tanpaiboon P. (2019). An Immune Tolerance Approach Using Transient Low-Dose Methotrexate in the ERT-Naïve Setting of Patients Treated with a Therapeutic Protein: Experience in Infantile-Onset Pompe Disease. Genet. Med..

[18] Parini R., De Lorenzo P., Dardis A., Burlina A., Cassio A., Cavarzere P., Concolino D., Della Casa R., Deodato F., Donati M.A. (2018). Long term clinical history of an Italian cohort of infantile onset Pompe disease treated with enzyme replacement therapy. Orphanet J. Rare Dis..

[19] Chien Y.H., Lee N.C., Thurberg B.L., Chiang S.C., Zhang X.K., Keutzer J., Huang A.C., Wu M.H., Huang P.H., Tsai F.J. (2009). Pompe disease in infants: Improving the prognosis by newborn screening and early treatment. Pediatrics.

[20] Chien Y.H., Lee N.C., Chen C.A., Tsai F.J., Tsai W.H., Shieh J.Y., Huang H.J., Hsu W.C., Tsai T.H., Hwu W.L. (2015). Long-term prognosis of patients with infantile-onset Pompe disease diagnosed by newborn screening and treated since birth. J. Pediatr..

[21] Kishnani P.S., Kronn D., Brassier A., Broomfield A., Davison J., Hahn S.H., Kumada S., Labarthe F., Ohki H., Pichard S. (2023). Safety and Efficacy of Avalglucosidase Alfa in Individuals with Infantile-Onset Pompe Disease Enrolled in the Phase 2, Open-Label Mini-COMET Study: The 6-Month Primary Analysis Report. Genet. Med..

[22] Kronn D., Davison J., Broomfield A., Brassier A., Labarthe F., Hahn S.H., Kumada S., Ohki H., Prakalapakorn S.G., Wilson C. (2025). The Mini-COMET Clinical Trial: Safety and Efficacy of Avalglucosidase Alfa after 97 Weeks of Treatment in Children with Infantile-Onset Pompe Disease Previously Treated with Alglucosidase Alfa. J. Pediatr..

[23] Fiumara A., Sapuppo A., Gasperini S., Crescitelli V., Sacchini M., Procopio E., Gragnaniello V., Burlina A. (2024). Avalglucosidase Alfa in Infantile-Onset Pompe Disease: A Snapshot of Real-World Experience in Italy. Mol. Genet. Metab. Rep..

[24] Chien Y.H., Chen H.A., Hsu R.H., Yeh C.H., Fang C.Y., Lee N.C., Hwu W.-L. (2025). Efficacy of transitioning from alglucosidase alfa to avalglucosidase alfa in infantile-onset Pompe disease: A single-center cohort analysis. Genet. Med..

[25] Chen L.R., Chen C.A., Chiu S.N., Chien Y.H., Lee N.C., Lin M.T., Hwu W.-L., Wang J.-K., Wu M.-H. (2009). Reversal of Cardiac Dysfunction after Enzyme Replacement in Patients with Infantile-Onset Pompe Disease. J. Pediatr..

[26] Gragnaniello V., Rizzardi C., Commone A., Gueraldi D., Maines E., Salviati L., Di Salvo G., Burlina A.B. (2023). Unusual Evolution of Hypertrophic Cardiomyopathy in Non-Compaction Myocardium in a Pompe Disease Patient. J. Clin. Med..

[27] Kishnani P.S., Diaz-Manera J., Toscano A., Clemens P.R., Ladha S., Berger K.I., Kushlaf H., Straub V., Carvalho G., Mozaffar T. (2023). Efficacy and Safety of Avalglucosidase Alfa in Patients With Late-Onset Pompe Disease After 97 Weeks: A Phase 3 Randomized Clinical Trial. JAMA Neurol..

[28] Kishnani P.S., Van Den Hout J.M.P., Hahn A., Kronn D., Chien Y.-H., Han M., Heuterman J., Sparks S., Glen C., Daba N. (2025). Insights into immunogenicity and therapeutic strategies to mitigate the immune response in infantile-onset Pompe disease: A comprehensive systematic literature review. Front. Immunol..

